# Synthesis, biological evaluation, and theoretical studies of 2-amino-3-cyano-4- (L-phenylalaninyl)quinolines: anticancer potential and EGFR inhibition

**DOI:** 10.55730/1300-0527.3758

**Published:** 2025-08-12

**Authors:** İlbilge Merve ŞENOL, Begüm Nurpelinx KARADUMAN ÖZKAN, İlhami ÇELİK, Ahmet Çağrı KARABURUN

**Affiliations:** 1 Department of Chemistry, Faculty of Science, Eskişehir Technical University, Eskişehir, Turkiye; 2 Department of Pharmaceutical Chemistry, Faculty of of Pharmacy, Anadolu University, Eskişehir, Turkiye

**Keywords:** 4-aminoquinoline, A549 cytotoxicity, MCF-7 cytotoxicity, EGFR inhibition, DFT analysis

## Abstract

Quinoline derivatives have garnered significant attention owing to their wide range of biological activities, particularly their anticancer potential. In this study, six novel 4-aminoquinoline derivatives incorporating a phenylalanine methyl ester moiety were synthesized and structurally characterized. The cytotoxic activities of the synthesized compounds were assessed against A549 and MCF-7 cancer cell lines, along with the noncancerous NIH3T3 fibroblast cell line. Compounds 4d and 4e displayed potent anticancer activity with low IC_50_ values, while exhibiting negligible toxicity toward normal cells. Moreover, these compounds exhibited moderate inhibitory activity against EGFR. Molecular docking studies were conducted to elucidate the binding modes of compounds 4d and 4e at the EGFR active site. To better elucidate their electronic structures and reactivity profiles, density functional theory (DFT) calculations were carried out to determine frontier molecular orbital energies, global reactivity descriptors, dipole moments, and molecular electrostatic potential (MEP) maps. Theoretical data were correlated with the experimental biological activities, revealing consistent trends, particularly among the most active compounds. Furthermore, theoretical NMR chemical shift calculations were performed for the synthesized compounds.

## 1. Introduction

Cancer is a disease characterized by uncontrolled cell proliferation, the spread of abnormal cells into surrounding tissues, and dysregulation of multiple genes, leading to tissue invasion and metastasis to distant organs, which, if left untreated, ultimately results in host mortality [1]. According to GLOBOCAN 2022 data provided by the World Health Organization (WHO), approximately 20 million cancer cases were reported globally. The most prevalent cancer types are lung, breast, and colorectal cancers. These data indicate that both global cancer incidence and related mortality are expected to rise substantially in the coming years [2]. With the continued increase in cancer incidence and mortality, the development of novel anticancer agents has emerged as a major focus in medicinal chemistry.

The epidermal growth factor receptor (EGFR) is a pivotal member of the receptor tyrosine kinase family, playing a crucial role in regulating cell proliferation, differentiation, and survival [3]. The EGFR-mediated tyrosine kinase signaling pathway is recognized as a critical mechanism in the initiation and progression of numerous solid tumors [1]. EGFR (HER1) and ERBB2 (HER2) are frequently implicated in breast, lung, ovarian, and other malignancies, whereas ERBB3 (HER3) is commonly associated with breast cancer, and ERBB4 (HER4) has been detected in both breast cancer and granulosa cell tumors [4]. Inhibition of EGFR is a central focus of targeted therapeutic strategies for numerous anticancer agents.

**Figure 1:**
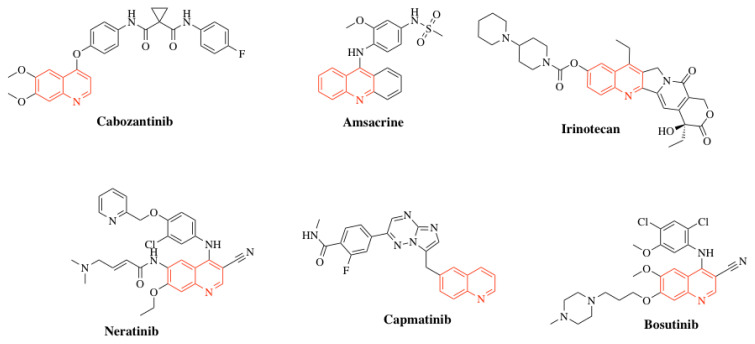
FDA-approved quinoline-based drugs.

Quinoline is an aromatic heterocyclic compound containing a nitrogen atom, consisting of a fused benzene and pyridine ring system. Also known as benzo[b]pyridine or 1-azanaphthalene, it exhibits weak basicity and hygroscopic characteristics. While it dissolves readily in organic solvents, its solubility in water remains limited [5]. The quinoline ring is a key structural motif in biologically active alkaloids such as berberine [6], camptothecin [7], and dictamine [8], which are isolated from flowering plants. In addition, synthetic quinoline derivatives have demonstrated a broad spectrum of biological activities, including antifungal [9], antibacterial [10], antioxidant [11], antiinflammatory [12], antimalarial [13], antiprotozoal [14], anti-HIV [15], and anticancer [16] properties in previous studies. The potential of quinoline derivatives, particularly in cancer therapy, represents a highly promising area of application. Numerous FDA-approved anticancer drugs feature the quinoline scaffold as a core structural component (Figure 1). Cabozantinib is a tyrosine kinase inhibitor targeting c-MET and VEGFR2, approved for the treatment of advanced renal cell carcinoma and medullary thyroid carcinoma [17]. Amsacrine is a potent antineoplastic agent containing a quinoline moiety, used in the treatment of malignant lymphomas and acute leukemia [18]. Irinotecan, a semisynthetic analog of camptothecin, is used in the treatment of metastatic colorectal carcinoma [19]. Neratinib is approved as a single-agent adjuvant for the treatment of early-stage breast cancer and, in combination with capecitabine, for the treatment of advanced or metastatic HER2-positive breast cancer [20]. Capmatinib is a selective mesenchymal–epithelial transition factor (MET) inhibitor approved for the treatment of nonsmall cell lung cancer [21]. Bosutinib is an orally administered Src/Abl tyrosine kinase inhibitor [18].

The presence of different substituents on the quinoline ring can profoundly influence the biological activities of these compounds. Quinoline derivatives bearing a cyano group at the C3 position play crucial roles in biological systems by inhibiting growth factor receptor activity, thereby modulating cell growth and proliferation [22]. In addition, amine groups at the C2 position of the quinoline ring have been reported to exhibit potent anticancer activities across multiple cancer types [23].

In this study, novel quinoline derivatives were designed based on literature precedents, incorporating an amine group at the C2 position, a cyano group at the C3 position, and a phenylalanine methyl ester at the C4 position. The synthesized compounds were evaluated for their potential anticancer activity against A549 (human lung adenocarcinoma) and MCF-7 (human breast adenocarcinoma) cell lines. Furthermore, the EGFR inhibitory profiles of the most active compounds were investigated. To elucidate the electronic structures and assess the chemical reactivity of the compounds, theoretical calculations were carried out using the DFT/B3LYP method with the 6–31G(d,p) basis set. Parameters including HOMO–LUMO energy levels, global reactivity descriptors, molecular electrostatic potential (MEP) maps, dipole moments, and theoretical NMR chemical shifts were obtained. These data were subsequently employed to elucidate potential correlations between the chemical properties and the observed biological effects of the compounds.

## 2. Materials and methods

### 2.1. Chemistry

#### 2.1.1. General experimental procedures

Melting points were determined using a Mettler Toledo MP90 apparatus (Mettler-Toledo International Inc., Colombus, OH, USA), and infrared (IR) spectra were recorded with a PerkinElmer Spectrum 100 FTIR spectrometer (PerkinElmer Inc., Waltham, MA, USA). Nuclear magnetic resonance (NMR) spectra were recorded on an Agilent Premium Compact spectrometer (Agilent Technologies Inc., Santa Clara, CA, USA) at 400 MHz for ^1^H NMR and 100 MHz for ^13^C NMR, a Bruker DPX 300 FT-NMR spectrometer (Bruker BioSpin GmbH, Rheinstetten, Germany) at 300 MHz for ^1^H NMR and 75 MHz for ^13^C NMR, and a JEOL ECZ500R spectrometer (JEOL Ltd., Tokyo, Japan) at 500 MHz for ^1^H NMR and 126 MHz for ^13^C NMR. High-resolution mass spectra (HRMS) were acquired using a Shimadzu LC-MS-IT-TOF hybrid mass spectrometer (Shimadzu Corporation, Kyoto, Japan). All solvents used for synthesis and purification were dried by standard methods to ensure their quality and suitability. The *N*-(2-aminobenzoyl)benzotriazole (1) derivatives used as starting materials were prepared according to a procedure previously reported by our research group [24].

#### 2.1.2. Synthesis of compounds

##### 2.1.2.1. Synthesis of 2-amino-4-hydroxyquinoline-3-carbonitriles (2a–f)

A mixture of *N*-(2-aminobenzoyl)benzotriazole (10 mmol), malononitrile (10 mmol), and tert-BuOK (10 mmol) was dissolved in 60 mL of dioxane and refluxed for 2 h. The reaction progress was monitored by thin-layer chromatography (TLC). Upon completion, the reaction mixture was cooled to room temperature. The solvent was removed under reduced pressure, and the residue was acidified to pH 2 with 2 N HCl. The precipitated solid was collected by filtration and purified by recrystallization from ethanol to afford the final crystalline product.

###### 2.1.2.1.1. 2-amino-4-hydroxyquinoline-3-carbonitrile (2a)

Off-white solid; yield: 70% (lit. 80% [25]). m.p. > 300 °C, (lit. m.p. > 360 °C [25]). FTIR (ν, cm^−1^): 3416 (O–H), 3329 (N–H_2_), 3218 (N–H_2_), 3075 (Ar–H), 2213 (C≡N), 1645 (C=N). ^1^H NMR (500 MHz, DMSO-d_6_): δ 11.16 (s, 1H, OH), 7.93 (d, *J* = 7.5 Hz, 1H, Ar–H), 7.57 (t, *J* = 7.6 Hz, 1H, Ar–H), 7.35 (d, *J* = 8.2 Hz, 1H, Ar–H), 7.23 (t, *J* = 7.5 Hz, 1H, Ar–H), 7.11 (s, 2H, NH_2_). ^13^C NMR (126 MHz, DMSO-d_6_): δ 175.02 (C4 quinoline), 156.19, 137.80, 132.40, 124.74, 122.98, 121.43, 117.08 (aromatic carbons), 116.98 (C≡N), 75.66 (C3 quinoline). HRMS (m/z): [M+H]^+^ calcd for C_10_H_7_N_3_O, 186.0662; found, 186.0651.

###### 2.1.2.1.2. 2-amino-4-hydroxy-7-methylquinoline-3-carbonitrile (2b)

White solid; yield: 54%; m.p. > 300 °C. FTIR (ν, cm^−1^): 3421 (O–H), 3166 (N–H_2_), 2972 (C–H, aliphatic), 2208 (C≡N), 1600 (C=N). ^1^H NMR (400 MHz, DMSO-d_6_): δ 11.11 (s, 1H, OH), 7.80 (d, *J* = 8.1 Hz, 1H, Ar–H), 7.11 (d, *J* = 8.1 Hz, 1H, Ar–H), 7.06 (s, 2H, NH_2_), 7.04 (s, 1H, Ar–H), 2.37 (s, 3H, CH_3_). ^13^C NMR (101 MHz, DMSO-d_6_): δ 175.39 (C4, quinoline), 156.71, 143.18, 138.37, 125.23, 124.84, 119.77, 117.66 (aromatic carbons), 117.08 (C≡N), 75.82 (C3, quinoline), 21.71 (CH_3_). HRMS (m/z): [M+H]^+^ calcd for C_11_H_9_N_3_O, 200.0818; found, 200.0792.

###### 2.1.2.1.3. 2-amino-7-fluoro-4-hydroxyquinoline-3-carbonitrile (2c)

White solid; yield: 60%; m.p. > 300 °C. FTIR (ν, cm^−1^): 3424 (O–H), 3334 (N–H_2_), 3226 (N–H_2_), 3109 (Ar–H), 2229 (C≡N), 1655 (C=N). ^1^H NMR (400 MHz, DMSO-d_6_): δ 11.24 (s, 1H, OH), 7.96 (dd, *J* = 8.8, 6.4 Hz, 1H, Ar–H), 7.30 (s, 2H, NH_2_), 7.17 (dd, *J* = 10.2, 2.5 Hz, 1H, Ar–H), 7.08 (td, *J* = 8.7, 2.5 Hz, 1H, Ar–H). ^13^C NMR (101 MHz, DMSO-d_6_): δ 174.22 (C4, quinoline), 164.25, 156.62, 139.53, 127.88, 118.44 (aromatic carbons), 116.90 (C≡N), 111.19, 103.28 (aromatic carbons), 75.48 (C3, quinoline). HRMS (m/z): [M+H]^+^ calcd for C_10_H_6_N_3_OF, 204.0568; found, 204.0558.

###### 2.1.2.1.4. 2-amino-7-chloro-4-hydroxyquinoline-3-carbonitrile (2d)

Off-white solid; yield: 72%; m.p. > 300 °C. FTIR (ν, cm^−1^): 3428 (O–H), 3331(N–H_2_), 3232 (N–H_2_), 2224 (C≡N), 1657 (C=N). ^1^H NMR (400 MHz, DMSO-d_6_): δ 11.26 (s, 1H, OH), 7.91 (dd, *J* = 8.8, 2.8 Hz, 1H, Ar–H), 7.45 (d, *J* = 2.2 Hz, 1H, Ar–H), 7.37 (s, 2H, NH_2_), 7.26 (dd, *J* = 8.5, 2.2 Hz, 1H, Ar–H). ^13^C NMR (101 MHz, DMSO-d_6_): δ 174.25 (C4, quinoline), 156.46, 138.89, 136.78, 126.90, 123.23, 120.23, 116.86 (aromatic carbons), 116.52 (C≡N), 75.86 (C3, quinoline). HRMS (m/z): [M+H]^+^ calcd for C_10_H_6_N_3_OCl, 220.0272; found, 220.0255.

###### 2.1.2.1.5. 2-amino-6-chloro-4-hydroxyquinoline-3-carbonitrile (2e)

Off-white solid; yield: 74%; m.p. 300 °C. FTIR (ν, cm^−1^): 3351 (O–H), 3200 (N–H_2_), 3132 (N–H_2_), 2233 (C≡N), 1663 (C=N). ^1^H NMR (400 MHz, DMSO-d_6_): δ 11.37 (s, 1H, OH), 7.85 (s, 1H, Ar–H), 7.62 (d, *J* = 7.0 Hz, 1H, Ar–H), 7.42 (d, *J* = 9.2 Hz, 1H, Ar–H), 7.32 (s, 2H, NH_2_). ^13^C NMR (101 MHz, DMSO-d_6_): δ 173.82 (C4, quinoline), 156.21, 136.66, 132.28, 127.42, 123.81, 122.70, 119.46 (aromatic carbons), 116.82 (C≡N), 75.96 (C3, quinoline). HRMS (m/z): [M+H]^+^ calcd for C_10_H_6_N_3_OCl, 220.0272; found, 220.0262.

###### 2.1.2.1.6. 2-amino-6-bromo-4-hydroxyquinoline-3-carbonitrile (2f)

Off-white solid; yield: 80%; m.p. > 300 °C. FTIR (ν, cm^−1^): 3348 (O–H), 3196 (N–H_2_), 3122 (N–H_2_), 2237 (C≡N), 1663 (C=N). ^1^H NMR (400 MHz, DMSO-d_6_): δ 11.35 (s, 1H, OH), 7.98 (d, *J* = 2.4 Hz, 1H, Ar–H), 7.73 (dd, *J* = 8.7, 2.4 Hz, 1H, Ar–H), 7.35 (d, *J* = 8.7 Hz, 1H, Ar–H), 7.30 (s, 2H, NH_2_). ^13^C NMR (101 MHz, DMSO-d_6_): δ 174.10 (C4, quinoline), 156.61, 137.37, 135.36, 127.28, 123.47, 120.07, 117.18 (aromatic carbons), 115.66 (C≡N), 76.36 (C3, quinoline). HRMS (m/z): [M+H]^+^ calcd for C_10_H_6_N_3_OBr, 263.9767; found, 263.9757.

##### 2.1.2.2. Synthesis of 2-amino-4-chloroquinoline-3-carbonitriles (3a–f)

To synthesize 2-amino-4-chloroquinoline-3-carbonitriles (3a–f), 2-amino-4-hydroxyquinoline-3-carbonitriles (2) (5 mmol) obtained from the previous step were reacted with phosphorus oxychloride (10 mL, excess) under a nitrogen atmosphere at 80 °C for 2–3 h. After completion, the reaction mixture was cooled to room temperature and carefully neutralized by the dropwise addition of 10% NaOH solution. Yellow precipitates (3a–f) formed during neutralization, which were collected by filtration and dried under vacuum to afford the final products.

###### 2.1.2.2.1. 2-amino-4-chloroquinoline-3-carbonitrile (3a)

Pale yellow solid; yield: 72%; m.p. 250 °C (decomp.). FTIR (ν, cm^−1^): 3410 (N–H_2_), 3390 (N–H_2_), 3118 (Ar–H), 2227 (C≡N), 1661 (C=N). ^1^H NMR (400 MHz, DMSO-d_6_): δ 7.96 (d, *J* = 8.4 Hz, 1H, Ar–H), 7.74 (t, *J* = 7.7 Hz, 1H, Ar–H), 7.56 (d, *J* = 8.5 Hz, 1H, Ar–H), 7.40 (t, *J* = 7.7 Hz, 1H, Ar–H), 7.22 (s, 2H, NH_2_). ^13^C NMR (101 MHz, DMSO-d_6_): δ 156.49 (C4, quinoline), 149.90, 148.61, 134.28, 126.64, 125.10, 124.38, 119.49 (aromatic carbons), 114.71 (C≡N), 95.87 (C3, quinoline). HRMS (m/z): [M+H]^+^ calcd for C_10_H_6_N_3_Cl, 204.0323; found, 204.0324.

###### 2.1.2.2.2. 2-amino-4-chloro-7-methylquinoline-3-carbonitrile (3b)

Yellow solid; yield: 63%; m.p. 244 °C (decomp.). FTIR (ν, cm^−1^): 3434 (N–H_2_), 3310 (N–H_2_), 3132 (Ar–H), 2924 (C–H, aliphatic), 2216 (C≡N), 1651 (C=N). ^1^H NMR (400 MHz, DMSO-d_6_): δ 7.83 (d, *J* = 8.5 Hz, 1H, Ar–H), 7.35 (s, 1H, Ar–H), 7.23 (dd, *J* = 8.5, 1.7 Hz, 1H, Ar–H), 7.17 (s, 2H, NH_2_), 2.44 (s, 3H, CH_3_). ^13^C NMR (101 MHz, DMSO-d_6_): δ 156.05 (C4, quinoline), 149.48, 147.61, 144.32, 125.90, 125.03, 124.19, 117.01 (aromatic carbons), 114.24 (C≡N), 94.10 (C3, quinoline), 21.32 (CH_3_). HRMS (m/z): [M+H]^+^ calcd for C_11_H_8_N_3_Cl, 218.0480; found, 218.0474.

###### 2.1.2.2.3. 2-amino-4-chloro-7-fluoroquinoline-3-carbonitrile (3c)

Yellow solid; yield: 79%; m.p. 240 °C (decomp.). FTIR (ν, cm^−1^): 3419 (N–H_2_), 3319 (N–H_2_), 3119 (Ar–H), 2225 (C≡N), 1663 (C=N). ^1^H NMR (400 MHz, DMSO-d_6_): δ 8.03 (dd, *J* = 8.9, 6.2 Hz, 1H, Ar–H), 7.40 (s, 2H, NH_2_), 7.31 (d, *J* = 8.6 Hz, 1H, Ar–H), 7.27 (s, 1H, Ar–H). ^13^C NMR (101 MHz, DMSO-d_6_): δ 164.10 (C7, quinoline), 156.76 (C4, quinoline), 148.31, 127.91, 116.47, 113.98 (aromatic carbons), 111.19 (C≡N), 110.93, 109.96 (aromatic carbons), 94.97 (C3, quinoline). HRMS (m/z): [M+H]^+^ calcd for C_10_H_5_N_3_FCl, 222.0229; found, 222.0222.

###### 2.1.2.2.4. 2-amino-4,7-dichloroquinoline-3-carbonitrile (3d)

Yellow solid; yield: 73%; m.p. 250 °C (decomp.). FTIR (ν, cm^−1^): 3421 (N–H_2_), 3318 (N–H_2_), 3155 (Ar–H), 2226 (C≡N), 1654 (C=N). ^1^H NMR (400 MHz, DMSO-d_6_): δ 7.94 (d, *J* = 8.8 Hz, 1H, Ar–H), 7.56 (d, *J* = 2.0 Hz, 1H, Ar–H), 7.44 (s, 2H, NH_2_), 7.39 (dd, *J* = 8.9, 2.0 Hz, 1H, Ar–H). ^13^C NMR (101 MHz, DMSO-d_6_): δ 156.79 (C4, quinoline), 149.96, 148.34, 138.59, 126.80, 124.71, 124.28, 117.87 (aromatic carbons), 114.05 (C≡N), 95.87 (C3, quinoline). HRMS (m/z): [M+H]^+^ calcd for C_10_H_5_N_3_Cl_2_, 237.9933; found, 237.9928.

###### 2.1.2.2.5. 2-amino-4,6-dichloroquinoline-3-carbonitrile (3e)

Yellow solid; yield: 76%; m.p. > 300 °C. FTIR (ν, cm^−1^): 3416 (N–H_2_), 3331 (N–H_2_), 3155 (Ar–H), 2228 (C≡N), 1673 (C=N). ^1^H NMR (400 MHz, DMSO-d_6_): δ 7.93 (d, *J* = 2.4 Hz, 1H, Ar–H), 7.74 (dd, *J* = 9.0, 2.4 Hz, 1H, Ar–H), 7.56 (d, *J* = 9.0 Hz, 1H, Ar–H), 7.39 (s, 2H, NH_2_). ^13^C NMR (101 MHz, DMSO-d_6_): δ 156.30 (C4, quinoline), 147.96, 134.02, 128.33, 128.02, 123.25, 119.77 (aromatic carbons), 113.98 (C≡N), 96.47 (C3, quinoline). HRMS (m/z): [M+H]^+^ calcd for C_10_H_5_N_3_Cl_2_, 237.9933; found, 237.9918.

###### 2.1.2.2.6. 2-amino-6-bromo-4-chloroquinoline-3-carbonitrile (3f)

Yellow solid; yield: 96%; m.p. > 300 °C. FTIR (ν, cm^−1^): 3418 (N–H_2_), 3329 (N–H_2_), 3132 (Ar–H), 2232 (C≡N), 1672 (C=N). ^1^H NMR (400 MHz, DMSO-d_6_): δ 8.07 (d, *J* = 2.3 Hz, 1H, Ar–H), 7.84 (dd, *J* = 9.0, 2.3 Hz, 1H, Ar–H), 7.49 (d, *J* = 8.9 Hz, 1H, Ar–H), 7.39 (s, 2H, NH_2_). ^13^C NMR (101 MHz, DMSO-d_6_): δ 156.38 (C4, quinoline), 148.28, 147.28, 136.53, 128.49, 126.36, 120.35, 116.02 (aromatic carbons), 114.00 (C≡N), 96.37 (C3, quinoline). HRMS (m/z): [M+H]^+^ calcd for C_10_H_5_N_3_ClBr, 281.9428; found, 281.9415.

##### 2.1.2.3. Synthesis of methyl (2-amino-3-cyanoquinolin-4-yl)-L-phenylalaninates (4a–f)

A mixture of 2-amino-4-chloroquinoline-3-carbonitriles (3a–f) (1 mmol), *L*-phenylalanine methyl ester hydrochloride (3 mmol), triethylamine (Et_3_N, 2 mL), dioxane (2 mL), and DMSO (0.5 mL) was heated at 50 °C under a nitrogen atmosphere for 12 h. The reaction progress was monitored by thin-layer chromatography (TLC). After completion, the reaction mixture was diluted with water and transferred to a separatory funnel. The aqueous layer was extracted with ethyl acetate (3 ×). The combined organic layers were washed successively with aqueous Na_2_CO_3_ and saturated NaCl solutions. The crude product was purified by column chromatography using EtOAc/hexane (1:2, v/v) as eluent.

###### 2.1.2.3.1. methyl (2-amino-3-cyanoquinolin-4-yl)-L-phenylalaninate (4a)

White solid; yield: 52%; m.p. 134–136 °C. FTIR (ν, cm^−1^): 3411 (N–H_2_), 3319 (N–H_2_), 3197 (N–H), 3087 (Ar–H), 2966 (C–H, aliphatic), 2217 (C≡N), 1739 (C=O), 1658 (C=N). ^1^H NMR (300 MHz, DMSO-d_6_): δ 8.10 (d, *J* = 8.5 Hz, 1H, Ar–H), 7.55 (d, *J* = 9.6 Hz, 1H, Ar–H), 7.49 (dd, *J* = 7.6, 2.0 Hz, 1H, Ar–H), 7.36 (dt, *J* = 8.0, 1.4 Hz, 2H, Ar–H), 7.32–7.26 (m, 1H, Ar–H), 7.24 (d, *J* = 1.4 Hz, 1H, Ar–H), 7.21 (d, *J* = 1.3 Hz, 1H, Ar–H), 7.37–7.34 (m, 2H, Ar–H, N–H), 6.29 (s, 2H, NH_2_), 5.29–5.21 (m, 1H, CH), 3.73 (s, 3H, OCH_3_), 3.36 (d, *J* = 1.9 Hz, 2H, CH_2_). ^13^C NMR (75 MHz, DMSO-d_6_): δ 171.82 (C=O), 157.56 (C2, quinoline), 154.37, (C4, quinoline), 148.75, 137.40, 131.89, 129.09, 128.45, 126.75, 126.00, 122.07, 121.15, 118.18 (aromatic carbons), 113.91 (C≡N), 71.96 (C3, quinoline), 58.66 (OCH_3_), 52.75 (CH), 37.34 (CH_2_). HRMS (m/z): [M+H]^+^ calcd for C_20_H_18_N_4_O_2_, 347.1503; found, 347.1509.

###### 2.1.2.3.2. methyl (2-amino-3-cyano-7-methylquinolin-4-yl)-L-phenylalaninate (4b)

White solid; yield: 51%; m.p. 214–216 °C. FTIR (ν, cm^−1^): 3462 (N–H_2_), 3364 (N–H_2_), 3204 (N–H), 3030 (Ar–H), 2957 (C–H, aliphatic), 2194 (C≡N), 1725 (C=O), 1663 (C=N). ^1^H NMR (400 MHz, DMSO-d_6_): δ 8.01 (d, *J* = 8.6 Hz, 1H, Ar–H), 7.49 (d, *J* = 9.1 Hz, 1H, Ar–H), 7.37 (d, *J* = 7.5 Hz, 2H, Ar–H), 7.25 (t, *J* = 7.5 Hz, 2H, Ar–H), 7.17 (d, *J* = 7.3 Hz, 1H, Ar–H), 7.11 (s, 1H, N–H), 7.00 (dd, *J* = 8.5, 1.8 Hz, 1H, Ar–H), 6.25 (s, 2H, NH_2_) 5.27–5.21 (m, 1H, CH), 3.75 (s, 3H, OCH_3_), 3.37 (s, 2H, CH_2_), 2.36 (s, 3H, CH_3_). ^13^C NMR (101 MHz, DMSO-d_6_): δ 172.32 (C=O), 158.16 (C2, quinoline), 154.74 (C4, quinoline), 149.39, 142.33, 137.86, 129.52, 128.87, 127.17, 125.81, 123.45, 122.36, 118.77 (aromatic carbons), 112.27 (C≡N), 71.93 (C3, quinoline), 59.04 (OCH_3_), 53.12 (CH), 37.81 (CH_2_), 21.62 (CH_3_). HRMS (m/z): [M+H]^+^ calcd for C_21_H_20_N_4_O_2_, 361.1659; found, 361.1663.

###### 2.1.2.3.3. methyl (2-amino-3-cyano-7-fluoroquinolin-4-yl)-L-phenylalaninate (4c)

White solid; yield: 58%; m.p. 146–148 °C. FTIR (ν, cm^−1^): 3467 (N–H), 3365 (N–H_2_), 3313 (N–H_2_), 3064 (Ar–H), 2954 (C–H, aliphatic), 2196 (C≡N), 1723 (C=O), 1645 (C=N). ^1^H NMR (300 MHz, DMSO-d_6_): δ 8.19 (dd, *J* = 9.3, 6.2 Hz, 1H, Ar–H), 7.64 (d, *J* = 9.1 Hz, 1H, Ar–H), 7.35 (d, *J* = 7.1 Hz, 2H, Ar–H), 7.24 (t, *J* = 7.4 Hz, 2H, Ar–H), 7.19–7.09 (m, 1H, N–H), 7.05 (td, *J* = 8.7, 2.7 Hz, 1H, Ar–H), 6.97 (dd, *J* = 10.9, 2.7 Hz, 1H, Ar–H), 6.48 (s, 2H, NH_2_), 5.27–5.24 (m, 1H, CH), 3.74 (s, 3H, OCH_3_), 3.33 (s, 2H, CH_2_). ^13^C NMR (75 MHz, DMSO-d_6_): δ 171.70 (C=O), 164.33 (C7, quinoline), 156.45 (C2, quinoline), 150.90, 137.30, 129.02, 128.40, 126.71, 125.19, 125.05 (aromatic carbons), 117.91(C≡N), 111.08, 110.21, 109.73 (aromatic carbons), 71.61 (C3, quinoline), 58.69 (OCH_3_), 52.70 (CH), 37.33 (CH_2_). HRMS (m/z): [M+H]^+^ calcd for C_20_H_17_N_4_O_2_F, 365.1408; found, 365.1406.

###### 2.1.2.3.4. methyl (2-amino-7-chloro-3-cyanoquinolin-4-yl)-L-phenylalaninate (4d)

White solid; yield: 62%; m.p. 139–141 °C. FTIR (ν, cm^−1^): 3420 (N–H), 3402 (N–H_2_), 3311 (N–H_2_), 3064 (Ar–H), 2956 (C–H, aliphatic), 2201 (C≡N), 1730 (C=O), 1650 (C=N). ^1^H NMR (300 MHz, DMSO-d_6_): δ 8.14 (d, *J* = 9.0 Hz, 1H, Ar–H), 7.67 (d, *J* = 9.0 Hz, 1H, Ar–H), 7.34 (d, *J* = 6.9 Hz, 2H, Ar–H), 7.30–7.25 (m, 1H, Ar–H), 7.24 (s, 1H, Ar–H), 7.22–7.11 (m, 3H, Ar–H, N–H), 6.51 (s, 2H, NH_2_), 5.28–5.20 (m, 1H, CH), 3.74 (s, 3H, OCH_3_), 3.35 (d, *J* = 3.7 Hz, 2H, CH_2_). ^13^C NMR (75 MHz, DMSO-d_6_): δ 171.62 (C=O), 158.52 (C2, quinoline), 154.24 (C4, quinoline), 149.80, 137.25, 136.51, 129.01, 128.38, 126.69, 124.41, 124.27, 121.12, 117.76 (aromatic carbons), 112.71 (C≡N), 72.16 (C3, quinoline), 58.67 (OCH_3_), 52.69 (CH), 37.31 (CH_2_). HRMS (m/z): [M+H]^+^ calcd for C_20_H_17_N_4_O_2_Cl, 381.1113; found, 381.1127.

###### 2.1.2.3.5. methyl (2-amino-6-chloro-3-cyanoquinolin-4-yl)-L-phenylalaninate (4e)

White solid; yield: 40%; m.p. 143–145 °C. FTIR (ν, cm^−1^): 3468 (N–H), 3366 (N–H_2_), 3309 (N–H_2_), 3093 (Ar–H), 2956 (C–H, aliphatic), 2196 (C≡N), 1724 (C=O), 1624 (C=N). ^1^H NMR (400 MHz, DMSO-d_6_): δ 8.29 (d, *J* = 2.3 Hz, 1H, Ar–H), 7.68 (d, *J* = 9.2 Hz, 1H, Ar–H), 7.52 (dd, *J* = 8.9, 2.2 Hz, 1H, Ar–H), 7.37 (d, *J* = 7.1 Hz, 2H, Ar–H), 7.33–7.24 (m, 3H, Ar–H), 7.20–7.14 (m, 1H, N–H), 6.48 (s, 2H, NH_2_), 5.29–5.25 (m, 1H, CH), 3.75 (s, 3H, OCH_3_), 3.37 (s, 2H, CH_2_). ^13^C NMR (101 MHz, DMSO-d_6_): δ 172.14 (C=O), 158.39 (C2, quinoline), 154.23 (C4, quinoline), 147.96, 137.78, 132.40, 129.51, 128.85, 128.36, 127.20, 125.77, 121.88, 118.20 (aromatic carbons), 115.24 (C≡N), 73.14 (C3, quinoline), 59.13 (OCH_3_), 53.18 (CH), 37.78 (CH_2_). HRMS (m/z): [M+H]^+^ calcd for C_20_H_17_N_4_O_2_Cl, 381.1113; found, 381.1118.

###### 2.1.2.3.6. methyl (2-amino-6-bromo-3-cyanoquinolin-4-yl)-L-phenylalaninate (4f)

White solid; yield: 61%; m.p. 173–175 °C. FTIR (ν, cm^−1^): 3467 (N–H_2_), 3448 (N–H_2_), 3366 (N–H), 3121 (Ar–H), 2957 (C–H, aliphatic), 2198 (C≡N), 1722 (C=O), 1641 (C=N). ^1^H NMR (300 MHz, DMSO-d_6_): δ 8.40 (s, 1H, Ar–H), 7.67 (d, *J* = 9.2 Hz, 1H, Ar–H), 7.61 (dd, *J* = 8.9, 2.1 Hz, 1H, Ar–H), 7.36 (d, *J* = 8.2 Hz, 2H, Ar–H), 7.29–7.19 (m, 3H, Ar–H), 7.19–7.12 (m, 1H, N–H), 6.44 (s, 2H, NH_2_), 5.30–5.22 (m, 1H, CH), 3.73 (s, 3H, OCH_3_), 3.37 (s, 2H, CH_2_). ^13^C NMR (75 MHz, DMSO-d_6_): δ 171.65 (C=O), 157.93 (C2, quinoline), 153.67 (C4, quinoline), 147.73, 137.28, 134.51, 129.03, 128.36, 128.08, 126.72, 124.44, 121.67, 117.70 (aromatic carbons), 115.40 (C≡N), 72.64 (C3, quinoline), 58.62 (OCH_3_), 50.08 (CH), 37.32 (CH_2_). HRMS (m/z): [M+H]^+^ calcd for C_20_H_17_N_4_O_2_Br, 425.0608; found, 425.0608.

### 2.2. Anticancer activity assays

#### 2.2.1. Cytotoxicity assay

The synthesized compounds were evaluated for anticancer activity against two cell lines: human lung adenocarcinoma (A549) and human breast adenocarcinoma (MCF-7). To assess selectivity, the compounds were also tested on healthy mouse embryonic fibroblast (NIH3T3) cells. The culture medium was supplemented with fetal calf serum, 100 IU/mL penicillin, and 100 μg/mL streptomycin. RPMI 1640 medium was used for the A549 and MCF-7 cell lines, whereas DMEM (Dulbecco’s modified eagle medium) was used for the NIH3T3 cell line. Doxorubicin was used as a reference drug to compare the efficacy of the tested compounds (4a–f). The cytotoxic effects of the compounds were measured using the MTT assay, as described previously [26–28].

#### 2.2.2. EGFR inhibition assay

The enzymatic activity of wild-type EGFR was measured using a luminescence-based kinase assay kit (BPS Bioscience, San Diego, CA, USA; cat. #40321), which employs the Kinase-Glo Max detection system (Promega Corporation, Madison, WI, USA).^1^ The assay was conducted in a 96-well plate format, and all samples, including controls, were tested in quadruplicate. Prior to the assay, the necessary components, including 5X kinase buffer, ATP (500 μM), and the protein tyrosine kinase (PTK) substrate Poly(Glu:Tyr, 4:1), were thawed and mixed with distilled water to prepare a master reaction mixture. The master mixture (25 μL per well) was dispensed into each well. Test wells received 5 μL of inhibitor solution, whereas control wells received 5 μL of inhibitor buffer without compound. The DMSO concentration was carefully maintained at or below 1% in the final reaction volume to avoid enzyme inhibition due to solvent effects. For the enzymatic reaction, recombinant wild-type EGFR was thawed on ice and diluted to a working concentration of 1 ng/μL in 1X kinase buffer. The reaction was initiated by adding 20 μL of the diluted enzyme to the designated wells, followed by incubation at 30 °C for 40 min. Wells designated as “Blank” received 1X kinase buffer instead of enzyme to account for background signal. After incubation, 50 μL of Kinase-Glo Max reagent was added to each well to terminate the reaction and generate the luminescent signal. The plate was incubated for an additional 15 min at room temperature, protected from light, after which luminescence was measured using a microplate reader. The luminescence intensity is inversely proportional to kinase activity, enabling quantification of EGFR inhibition by the test compounds.

### 2.3. Molecular docking studies

A structure-based in silico docking approach was applied to identify the possible interaction and binding sites of compounds 4d and 4e on EGFR. Protein–ligand interaction analysis was performed using the crystal structure of EGFR (PDB ID: 1M17) [29]. The crystal structure was prepared using the “Protein Preparation Wizard” protocol in Schrödinger Suite 2020 Update 3 [30], after which it was ready for docking studies. Bond lengths were optimized using the OPLS-2005 force field, and the partial charges on amino acid residues were automatically assigned according to the specified environmental conditions. The ligands used for molecular docking studies were prepared using the LigPrep 3.8 module [31]. The receptor grid was generated using Glide 7.1 [32], and docking studies were performed in single-precision (SP) mode within the same module.

### 2.4. Computational methods

Quantum chemical calculations were performed on the synthesized compounds (4a–f) using density functional theory (DFT) with the hybrid functional B3LYP, as implemented in the Gaussian 09 software package [33]. Molecular geometry optimizations in the gas phase were carried out using the semiempirical PM6 parameterization and DFT/B3LYP with the 3–21G and 6–31G(d,p) basis sets, respectively. After optimization, frequency calculations were performed at the same theoretical level in both the gas phase and the DMSO solvent phase. Excited-state energy calculations were performed using Time-Dependent DFT (TD-DFT) with the B3LYP functional and the 6–31G(d,p), basis set to obtain HOMO–LUMO energies. HOMO and LUMO orbital images were generated using the GaussView 5.0 interface [34], and the corresponding energy values were obtained with GaussSum 3.0. NMR chemical shift calculations were performed at the DFT/B3LYP/6–31G(d,p) level using the gauge-independent atomic orbital (GIAO) method [35] and the conductor-like polarizable continuum solvent model (CPCM) [36] with DMSO as solvent. The HOMO–LUMO band gaps, global reactivity descriptors, dipole moments, MEP maps, and ^1^H and ^13^C NMR chemical shifts of the synthesized compounds (4a–f) were obtained from the DFT output files.

## 3. Results and discussion

The Scheme illustrates the general synthetic pathway. The synthesis of 2-amino-4-hydroxyquinoline-3-carbonitriles (2) was accomplished by reacting *N*-(2-aminobenzoyl)benzotriazoles (1) with malononitrile in the presence of tert-BuOK. In the next step, the hydroxyl group (OH) at the 4-position of the resulting 2-amino-4-hydroxyquinoline-3-carbonitriles was replaced with a chlorine atom (Cl) using POCl_3_, affording 2-amino-4-chloroquinoline-3-carbonitriles (3). Subsequently, reaction of these intermediates with *L*-phenylalanine methyl ester hydrochloride in a mixture of dioxane and DMSO, using Et_3_N as base, afforded methyl (2-amino-3-cyanoquinolin-4-yl)-*L*-phenylalaninates (4a–f).

### 3.1. Characterization of synthesized compounds

#### 3.1.1. Characterization of 2-amino-4-hydroxyquinoline-3-carbonitriles (2a–f)

The ^1^H NMR spectra of compounds 2a–f showed a characteristic singlet at approximately 11 ppm, corresponding to the hydroxyl (OH) proton. A singlet between 7.0 and 7.5 ppm, integrating for two protons, was assigned to the amino (NH_2_) group. Signals in the range of 7–8 ppm were attributed to aromatic ring protons. For compound 2b, the methyl (CH_3_) protons resonated at approximately 2 ppm.

In the ^13^C NMR spectra of compounds 2a–f, the signal at 175 ppm was assigned to the carbon atom bearing the hydroxyl (OH) group. Aromatic carbons appeared between 160 and 117 ppm, whereas the nitrile (CN) carbon resonated near 116 ppm. The carbon adjacent to the −CN group resonated at approximately 75 ppm, showing an upfield shift attributed to the anisotropic effect of the nitrile group. The methyl (CH_3_) carbon in compound 2b appeared at approximately 20 ppm.

In the IR spectra of compounds 2a–f, a strong band near 2200 cm^−1^ was assigned to the C≡N stretching vibration, whereas a doublet around 3300 cm^−1^ corresponded to the N–H stretching vibrations of the amino (NH_2_) group. These spectral features confirm the successful formation of the quinoline ring system and are consistent with previously reported data for compound 2a [25].

#### 3.1.2. Characterization of 2-amino-4-chloroquinoline-3-carbonitriles (3a–f)

The ^1^H NMR spectra of compounds 3a–f showed the disappearance of the singlet at 11 ppm, which was previously observed in the spectra of 2-amino-4-hydroxyquinoline-3-carbonitriles (2a–f). Similarly, the absence of the signal at 175 ppm in the ^13^C NMR spectra of 3a–f indicates that the hydroxyl group has been replaced by a chlorine atom (Cl). These observations confirm the substitution of the hydroxyl group at the 4-position of the quinoline ring with chlorine.

#### 3.1.3. Characterization of methyl (2-amino-3-cyanoquinolin-4-yl)-L-phenylalaninates (4a–f)

**Figure 2:**
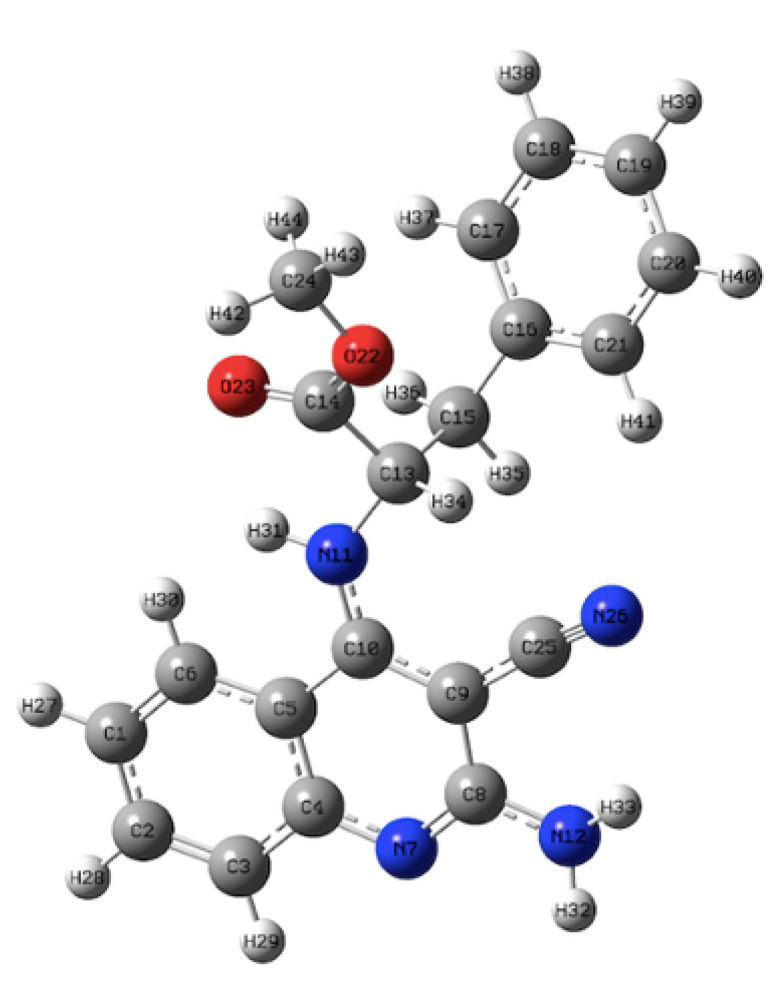
3D structure of compound 4a

The 3D structure of compound 4a is shown in Figure 2, and the NMR spectra of compounds 4a–f are provided in the supplementary data. The ^1^H NMR spectra of compounds 4a–f exhibited distinct signals in characteristic regions. Signals in the 7–8 ppm range were assigned to protons of the quinoline and phenyl rings, as well as the −NH proton (H-31). A singlet signal in the 6.0–6.5 ppm range was attributed to the NH_2_ protons (H-32, H-33). A multiplet signal at approximately 5 ppm was assigned to the −CH− proton (H-34). Additionally, a singlet at approximately 3.7 ppm corresponded to the methoxy protons (OCH_3_), whereas a signal at around 3.3 ppm was assigned to the −CH_2_− protons (H-35, H-36). The ^13^C NMR spectra of compounds 4a–f exhibited a signal at approximately 171 ppm corresponding to the carbonyl carbon, along with signals in the 164–118 ppm range assigned to aromatic carbons. The signal at 113–115 ppm was assigned to the nitrile (C≡N) carbon (C-25), whereas the signal at around 70 ppm corresponded to C-9. Signals in the 58–37 ppm range were attributed to aliphatic carbons [37]. The IR spectra of compounds 4a–f exhibited a characteristic band at approximately 2200 cm^−1^ corresponding to the C≡N stretching vibration. The double band in the 3300–3400 cm^−1^ range was assigned to N–H stretching of the NH_2_ group, whereas the single band corresponded to N–H stretching of the −NH group. A sharp peak at 1725 cm^−1^ corresponded to the ester C=O stretching vibration [38].

### 3.2. Anticancer activity evaluation

#### 3.2.1. Cytotoxicity results

The cytotoxic effects of the compounds were evaluated using a 24 h MTT assay, and the results are summarized in Table 1. Compounds 4d and 4e exhibited significant activity against A549 and MCF-7 cell lines, with IC_50_ values below 10 μM. For the A549 cell line, compound 4d showed an IC_50_ of 3.317 ± 0.142 μM, whereas compound 4e displayed an IC_50_ of 4.648 ± 0.199 μM. In the MCF-7 cell line, IC_50_ values were 7.711 ± 0.217 μM for 4d and 6.114 ± 0.272 μM for 4e. In the NIH3T3 cell line, compounds 4d and 4e were nontoxic at concentrations effective against the A549 and MCF-7 cell lines.

#### 3.2.2. EGFR inhibition results

The in vitro EGFR inhibitory potential of the two active compounds was evaluated according to the kit protocol [29]. The results are presented in Table 2. Compounds 4d and 4e exhibited inhibitory activity against EGFR, with IC_50_ values of 0.069 ± 0.0027 μM and 0.058 ± 0.0026 μM, respectively. Their activity was moderate compared to the reference inhibitor erlotinib (IC_50_ = 0.002 ± 0.0001 μM).

### 3.3. Molecular docking results

**Figure 3:**
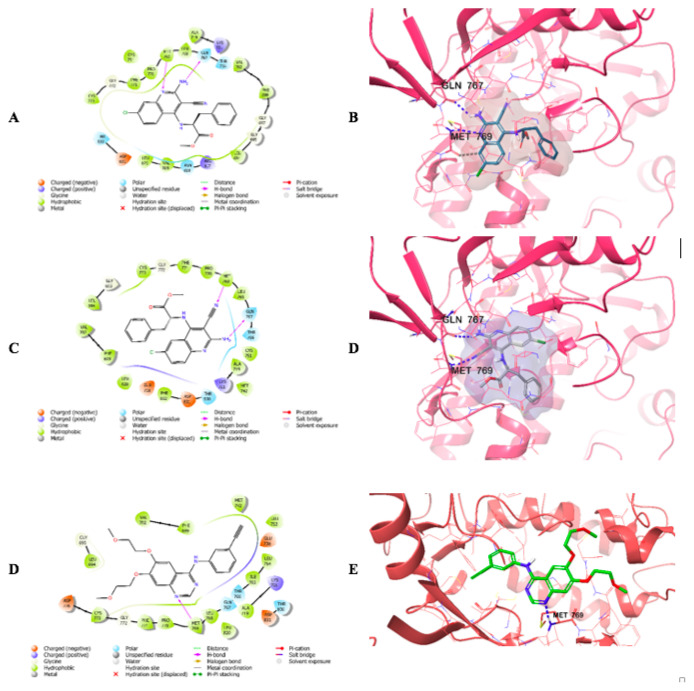
2D (A, C, E) and 3D (B, D, F) interaction views of compounds 4d, 4e, and erlotinib in the EGFR active site.

Based on the activity results, compounds 4d and 4e exhibited the highest EGFR inhibition among the synthesized derivatives. Hence, molecular docking studies were performed to investigate the potential interactions and binding sites of compounds 4d and 4e, along with the reference compound erlotinib, with EGFR. Figure 3 illustrates the docking poses of erlotinib (E, F), compound 4d (A, B), and compound 4e (C, D) with EGFR. The corresponding binding scores are presented in Table 3. Docking analysis of erlotinib showed that its quinazoline ring formed a hydrogen bond with Met769 through a nitrogen atom. The docking poses of compounds 4d and 4e revealed hydrogen bond interactions with Met769 via the nitrogen atom of their quinoline ring. Additionally, the primary amino group at the 2-position of the quinoline ring contributed significantly to polar interactions. Hydrogen bonds were established between the primary amine group and Gln767. For compound 4d, an aromatic hydrogen bond was observed between the quinoline ring and the carbonyl group of Met769. Overall, the docking studies showed that compounds 4d and 4e exhibited binding interactions similar to those of erlotinib and docked strongly into the EGFR active site, with additional binding contributions. These findings highlight the potent EGFR inhibitory profile of compounds 4d and 4e.

### 3.4. DFT analysis

#### 3.4.1. Frontier molecular orbital analysis

**Figure 4:**
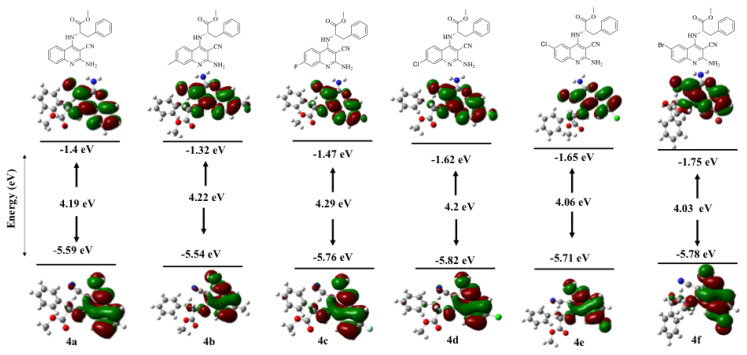
HOMO–LUMO orbitals and energy gaps (E_gap_) of compounds 4a–f in the gas phase.

The highest occupied molecular orbital (HOMO) energy level reflects the ionization potential of a molecule and indicates its electron-donating ability. Conversely, the lowest unoccupied molecular orbital (LUMO) energy corresponds to the electron affinity, representing the molecule’s electron-accepting capacity. The energy gap (*E*_gap_) is defined as the energy difference between the HOMO and LUMO levels. This gap is a key parameter for understanding molecular stability and reactivity. A smaller energy gap indicates higher molecular reactivity. In contrast, a larger energy gap implies greater stability and lower reactivity [39]. Previous studies have shown that HOMO and LUMO energy levels are correlated with biological activity [40]. HOMO–LUMO analyses of the synthesized compounds were carried out using the TD-DFT/B3LYP method with the 6–31G(d,p) basis set, and the results are summarized in Table 4 and Figure 4. Examination of the HOMO–LUMO band gaps revealed that both orbitals were predominantly localized on the quinoline ring. The stability order of the compounds in the gas phase was 4c > 4b > 4d > 4a > 4e > 4f, whereas in DMSO it was 4c > 4b > 4a = 4d > 4e = 4f. These findings indicate that compound 4f is the most reactive in the gas phase, whereas compounds 4e and 4f exhibit the highest reactivity in DMSO.

#### 3.4.2. Global reactivity descriptors

Global chemical reactivity descriptors are widely used to predict molecular behavior. Quantum chemical reactivity parameters—including ionization potential (I = −*E*_HOMO_), electron affinity (A = −*E*_LUMO_), chemical potential (μ = −(I + A) / 2), global hardness (η = (I – A) / 2), global softness (S = 1/η), electronegativity (χ = −μ), and electrophilicity (ω = μ^2^/2η)—were calculated to provide insights into molecular properties and reactivity patterns (Table 3). The ionization potential (I) represents the minimum energy required to remove an electron from a molecule in the gas phase [41], whereas the electron affinity (A) indicates the energy released upon electron addition [42]. In the gas phase, compound 4b exhibited the lowest ionization potential (5.54 eV), whereas compound 4d showed the highest (5.82 eV). In DMSO, compound 4a showed the lowest ionization potential (5.77 eV), whereas 4d had the highest (5.91 eV). In the gas phase, compound 4b had the lowest electron affinity (1.32 eV), whereas 4f had the highest (1.75 eV). In DMSO, compound 4b again showed the lowest electron affinity (1.49 eV), while 4e and 4f had the highest (1.73 eV). These findings highlight that electron-donating and electron-accepting tendencies are significantly influenced by substituent electronic effects. Chemical hardness (η) and softness (S) describe molecular stability and reactivity. Hardness reflects resistance to electronic changes and stability, whereas softness indicates susceptibility to charge transfer and higher reactivity [43]. Molecules with greater softness are generally more reactive, whereas those with higher hardness are more stable. In both gas and DMSO phases, compounds 4f and 4e showed the highest softness, indicating greater reactivity. Compound 4c exhibited the lowest softness, indicating greater stability. This trend was consistent across both phases, underscoring the role of bromine and chlorine substituents in enhancing softness and reactivity. Electronegativity (*X*) values indicated that the 6-Br-substituted compound 4e in the gas phase and the 7-Cl-substituted compound 4d in DMSO exhibited a stronger ability to attract bonding electrons than the other compounds. In the gas phase, the electrophilicity index (ω) order was 4b < 4a < 4c < 4d < 4e < 4f, with 4f showing the highest electrophilic character and 4b the lowest. In DMSO, the order was 4b < 4a < 4c < 4d < 4e = 4f, with 4e and 4f exhibiting identical values and stronger electrophilic character than the others. The negative chemical potential (μ) values indicate that these compounds are not decomposable into their constituent elements [44]. All compounds exhibited negative chemical potential (μ) values. Compounds 4d and 4e, which exhibited biological activity, had lower HOMO–LUMO gaps and higher softness values, indicating higher reactivity and greater ability to interact with biological targets [45]. Furthermore, their high electrophilicity indices align with previous reports indicating that more electrophilic molecules often display greater biological activity [46]. Although a strict correlation was not observed across all compounds, the overall trends support the relevance of global reactivity descriptors in predicting biological activity.

#### 3.4.3. Dipole moments

The dipole moment can be correlated with biological activity. Higher biological activity is generally attributed to the enhanced cellular interactions of more polar molecules [47]. However, the dipole moment also reflects the hydrophobic or hydrophilic properties of a compound, an important parameter governing membrane permeability. In particular, lipophilicity is known to play a key role in cellular uptake. Studies have demonstrated that a lower dipole moment, corresponding to increased lipophilicity, is often associated with enhanced anticancer efficacy [48]. DFT/B3LYP/6–31G(d,p) calculations revealed that the dipole moment values of the synthesized compounds (4a–f) in the gas phase ranged from 2.3153 to 3.8760 D. In DMSO, these values increased to 2.7438–4.8934 D. The use of DMSO, a polar solvent, resulted in higher dipole moment values. Notably, among the two compounds active in cytotoxicity assays, compound 4d exhibited the highest dipole moment (4.8934 D in DMSO), whereas compound 4e displayed the lowest (2.7438 D). This contrast clearly demonstrates a lack of correlation between dipole moment and anticancer activity (Table 5).

#### 3.4.4. Molecular electrostatic potential (MEP) maps

**Figure 5:**
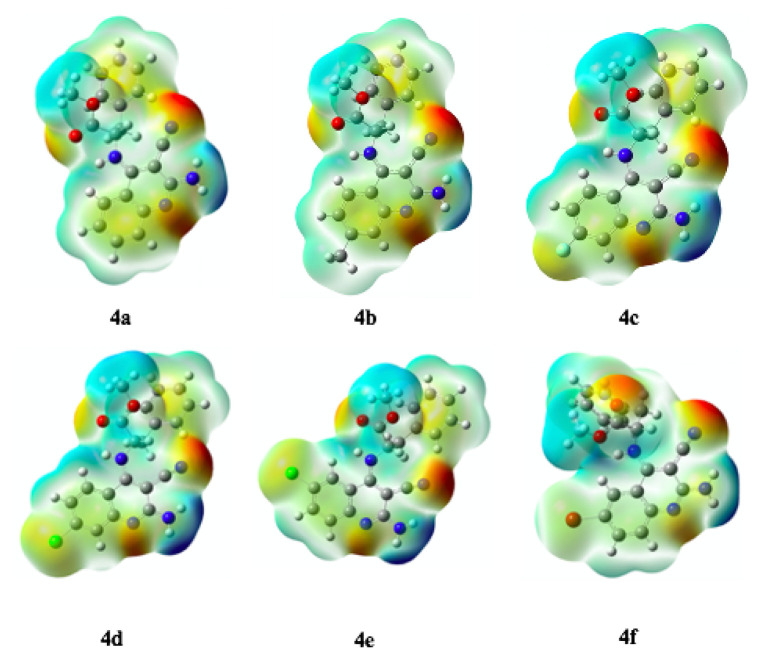
Molecular electrostatic potential (MEP) maps of the synthesized compounds (4a–4f).

Molecular electrostatic potential (MEP) maps are widely employed to identify molecular interactions, including hydrogen bonding, drug–receptor interactions, enzyme–substrate interactions, and reactive regions prone to electrophilic or nucleophilic attack [49]. The MEPs of the synthesized compounds (4a–f) were calculated at the DFT/B3LYP/6–31G(d,p) level. According to the MEP maps, the most electron-rich regions correspond to the −CN group at the 3-position of the quinoline ring (red area), whereas the most electron-deficient regions correspond to the −NH_2_ protons at the 2-position (blue area) (Figure 5).

#### 3.4.5. NMR calculations

**Figure 6:**
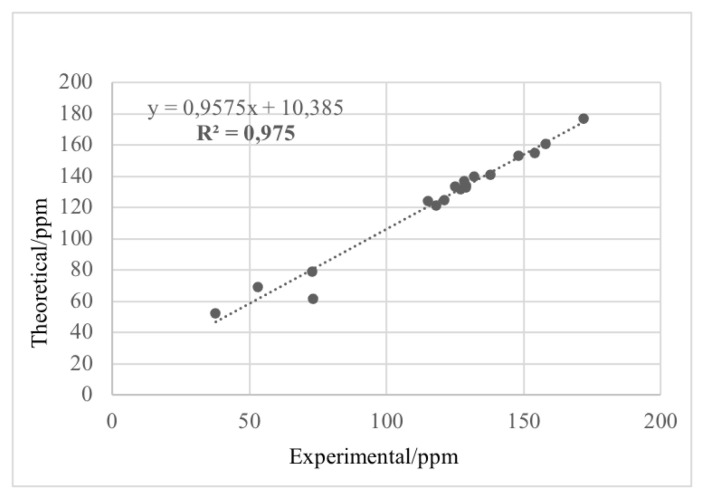
Correlation graph of experimental and calculated ^13^C NMR data for compound 4e.

**Figure 7:**
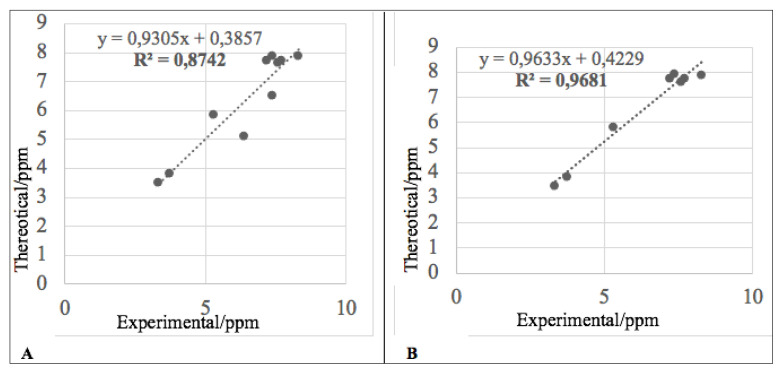
Correlation graph of experimental and calculated ^1^H NMR data for compound 4e.

NMR calculations of the synthesized compounds were performed at the DFT/B3LYP/6–31G(d,p) level using the CPCM model in DMSO. The correlation between experimental and calculated chemical shifts is shown in the correlation graph. The R^2^ values obtained from the correlation graphs for the ^13^C NMR and ^1^H NMR data of compound 4e were 0.975 (Figure 6) and 0.8742 (Figure 7A), respectively. These values confirm the consistency between experimental and calculated NMR data. The slight deviation observed in the ^1^H NMR correlation graph was attributed to the −NH and −NH_2_ protons. These protons are highly sensitive to external factors such as temperature and solvent effects. Upon removal of these points from the graph, the R^2^ value increased to 0.9681 (Figure 7B).

## 4. Conclusion

In this study, six novel quinoline derivatives were synthesized in yields of 51%–62% and structurally characterized by IR, ^1^H NMR, ^13^C NMR, and HRMS spectroscopy. The cytotoxic activities were evaluated against A549, MCF-7, and NIH3T3 (healthy) cell lines. Compounds 4d and 4e showed significant cytotoxicity against A549 and MCF-7 cells, with no detectable toxicity toward NIH3T3 cells at effective concentrations. In A549 cells, compounds 4d and 4e had IC_50_ values of 3.317 ± 0.142 μM and 4.648 ± 0.199 μM, respectively. In MCF-7 cells, the IC_50_ values were 7.711 ± 0.217 μM for 4d and 6.114 ± 0.272 μM for 4e. The EGFR inhibitory activities of the cytotoxic compounds were also investigated. Compound 4d showed an IC_50_ of 0.069 ± 0.0027 μM, while compound 4e showed 0.058 ± 0.0026 μM. Compared with the reference drug erlotinib, both compounds displayed moderate activity. Molecular docking studies further confirmed that compounds 4d and 4e bind strongly to the EGFR active site, supporting their profiles as potent EGFR inhibitors. The HOMO–LUMO energy gaps, global reactivity descriptors, dipole moments, and molecular electrostatic potential (MEP) maps were calculated at the DFT/B3LYP/6–31G(d,p) level to gain deeper insight into their electronic structures and reactivity profiles. The resulting HOMO–LUMO analyses and global descriptors were further correlated with the observed biological activities, revealing notable trends, particularly among the active compounds. Moreover, the strong correlation (R^2^ close to 1) between theoretical and experimental NMR shifts for compound 4e confirmed the accuracy of the computational method.

## Supplementary Information



## Figures and Tables

**Scheme:**
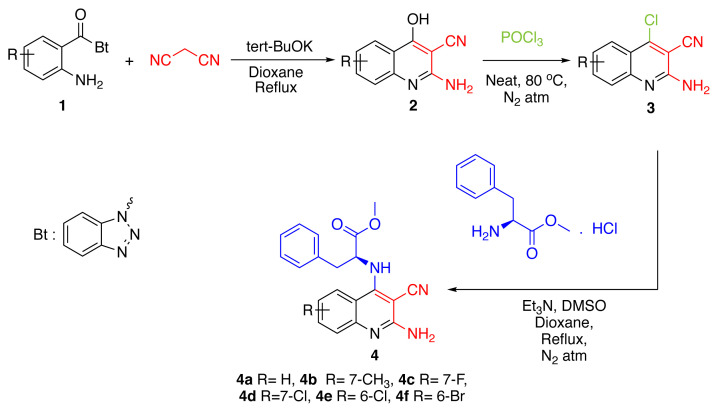
General synthetic route.

**Table 1:** IC_50_ values (μM) of the compounds in A549, MCF-7, and NIH3T3 cell lines.

Compound	IC_50_ (μM)		
	A549	MCF7	NIH3T3
**4a**	>100	>100	>100
**4b**	>100	>100	>100
**4c**	23.610 ± 0.523	27.236 ± 1.013	30.458 ± 1.320
**4d**	**3.317 ± 0.142**	**7.711 ± 0.217**	>100
**4e**	**4.648 ± 0.199**	**6.114 ± 0.272**	56.341 ± 2.057
**4f**	>100	>100	>100
**Doxorubicin**	10.985 ± 0.247	1.940 ± 0.084	>100

**Table 2:** IC_50_ values (μM) of selected compounds against EGFR.

Compound	EGFR IC_50_ (μM)
**4d**	0.069 ± 0.0027
**4e**	0.058 ± 0.0026
**Erlotinib**	0.002 ± 0.0001

**Table 3:** Binding scores (kcal/mol) of compounds 4d, 4e, and erlotinib.

Compound	Docking score	Glide gscore	Glide emodel

**4d**	−5.141	−5.141	−49.557
**4e**	−5.801	−5.801	−52.195
**Erlotinib**	−8.831	−8.831	−87.435

**Table 4:** Global chemical reactivity descriptors of the synthesized compounds (4a–f).

Compound	Phase	HOMO (eV)	LUMO (eV)	Egap (eV)	Ionization potential (*I*)	Electron affinity (*A*)	μ (eV)	η	S	χ (eV)	ω (eV)
**4a**	**Gas**	−5.59	−1.4	4.19	5.59	1.4	−3.495	2.095	0.477	3.495	2.915
	**DMSO**	−5.77	−1.57	4.2	5.77	1.57	−3.67	2.1	0.476	3.67	3.207
**4b**	**Gas**	−5.54	−1.32	4.22	5.54	1.32	−3.43	2.11	0.474	3.43	2.788
	**DMSO**	−5.72	−1.49	4.23	5.72	1.49	−3.605	2.115	0.473	3.605	3.072
**4c**	**Gas**	−5.76	−1.47	4.29	5.76	1.47	−3.615	2.145	0.466	3.615	3.046
	**DMSO**	−5.87	−1.57	4.3	5.87	1.57	−3.72	2.15	0.465	3.72	3.218
**4d**	**Gas**	−5.82	−1.62	4.2	5.82	1.62	−3.72	2.1	0.476	3.72	3.295
	**DMSO**	−5.91	−1.71	4.2	5.91	1.71	−3.81	2.1	0.476	3.81	3.456
**4e**	**Gas**	−5.71	−1.65	4.06	5.71	1.65	−3.68	2.03	0.493	3.68	3.336
	**DMSO**	−5.81	−1.73	4.08	5.81	1.73	−3.77	2.04	0.49	3.77	3.484
**4f**	**Gas**	−5.78	−1.75	4.03	5.78	1.75	−3.765	2.015	0.496	3.765	3.517
	**DMSO**	−5.81	−1.73	4.08	5.81	1.73	−3.77	2.04	0.49	3.77	3.484

**Table 5:** Dipole moment values of the synthesized compounds (4a–4f).

Compound	Dipole moment (Debye) Gas	Dipole moment (Debye) DMSO

**4a**	2.7640	4.1342
**4b**	2.7389	3.9428
**4c**	3.4257	4.4209
**4d**	3.8760	4.8934
**4e**	2.3153	2.7438
**4f**	2.4010	3.2061

## Footnotes

1 Max detection system. https://Bpsbioscience.Com/Egfr-Kinase-Assay-Kit [accessed 06.06.2024]
