# Supplementary material for: Synthesis, biological evaluation, and theoretical studies of 2-amino-3-cyano-4- (L-phenylalaninyl)quinolines: anticancer potential and EGFR inhibition
Source: Turk J Chem. 2025 Aug 12;49(5):616–31. doi: 10.55730/1300-0527.3758 (PMC12604922; doi:10.55730/1300-0527.3758)
Supplement: Supplementary file 1 [file tjc-49-05-616-Ilbilge_Merve_senol_supplemental_data.docx]

Synthesis, biological evaluation and theoretical calculation of 2-amino-3-cyano-4-phenylalaninquinolines: anticancer potential and EGFR inhibition

İlbilge Merve ŞENOL^1*^, Begüm Nurpelin SAĞLIK ÖZKAN^2^, İlhami ÇELİK^1^, and Ahmet Çağrı KARABURUN^2^

^1^Department of Chemistry, Faculty of Science, Eskisehir Technical University, 26470 Eskişehir, Turkey

^2^Department of Pharmaceutical Chemistry, Faculty of Pharmacy, Anadolu University, 26470 Eskişehir, Turkey

**Figure S1.** *^1^H NMR Spectrum of 2-Amino-4-hydroxyquinoline-3-carbonitrile (2a)*

**Figure S2.** *^13^C NMR Spectrum of* *^1^H NMR Spectrum of 2-Amino-4-hydroxyquinoline-3-carbonitrile (2a)*

**Figure S3.** *^1^H NMR Spectrum of 2-Amino-4-hydroxy-7-methylquinoline-3-carbonitrile (2b)*

**Figure S4***. ^13^C NMR Spectrum of 2-Amino-4-hydroxy-7-methylquinoline-3-carbonitrile (2b)*

**Figure S5.** *^1^H NMR Spectrum of 2-Amino-7-fluoro-4-hydroxyquinoline-3-carbonitrile (2c)*

**Figure S6.** *^13^C NMR Spectrum of 2-Amino-7-fluoro-4-hydroxyquinoline-3-carbonitrile (2c)*

**Figure S7.** *^1^H NMR Spectrum of 2-Amino-7-chloro-4-hydroxyquinoline-3-carbonitrile (2d)*

**Figure S8.** *^13^C NMR Spectrum of 2-Amino-7-chloro-4-hydroxyquinoline-3-carbonitrile (2d)*

**Figure S9.** *^1^H NMR Spectrum of 2-Amino-6-chloro-4-hydroxyquinoline-3-carbonitrile (2e)*

**Figure S10.** *^13^C NMR Spectrum of 2-Amino-6-chloro-4-hydroxyquinoline-3-carbonitrile (2e)*

**Figure S11**. *^1^H NMR Spectrum of 2-Amino-6-bromo-4-hydroxyquinoline-3-carbonitrile (2f)*

**Figure S12.** *^13^C NMR Spectrum of 2-Amino-6-bromo-4-hydroxyquinoline-3-carbonitrile (2f)*

**Figure S13.** *^1^H NMR Spectrum of 2-Amino-4-chloroquinoline-3-carbonitrile (3a)*

**Figure S14.** *^13^C NMR Spectrum of 2-Amino-4-chloroquinoline-3-carbonitrile (3a)*

**Figure S15.** *^1^H NMR Spectrum of 2-Amino-4-chloro-7-methylquinoline-3-carbonitrile (3b)*

**Figure S16.** *^13^C NMR Spectrum of 2-Amino-4-chloro-7-methylquinoline-3-carbonitrile (3b)*

**Figure S17.** *^1^H NMR Spectrum of 2-Amino-4-chloro-7-fluoroquinoline-3-carbonitrile (3c)*

**Figure S18.** *^13^C NMR Spectrum of 2-Amino-4-chloro-7-fluoroquinoline-3-carbonitrile (3c)*

**Figure S19.** *^1^H NMR Spectrum of 2-Amino-4,7-dichloroquinoline-3-carbonitrile (3d)*

**Figure S20.** *^13^C NMR Spectrum of 2-Amino-4,7-dichloroquinoline-3-carbonitrile (3d)*

**Figure S21.** *^1^H NMR Spectrum of 2-Amino-4,6-dichloroquinoline-3-carbonitrile (3e)*

**Figure S22.** *^13^C NMR Spectrum of 2-Amino-4,6-dichloroquinoline-3-carbonitrile (3e)*

**Figure S23.** *^1^H NMR Spectrum of 2-Amino-6-bromo-4-chloroquinoline-3-carbonitrile (3f)*

**Figure S24.** *^13^C NMR Spectrum of 2-Amino-6-bromo-4-chloroquinoline-3-carbonitrile (3f)*

**Figure S25.** *^13^H NMR Spectrum methyl (2-amino-3-cyanoquinolin-4-yl)-L-phenylalaninate (4a)*

**Figure S26.** ^13^C NMR Spectrum of methyl (2-amino-3-cyanoquinolin-4-yl)-L-phenylalaninate (4a)

**Figure S27.** ^1^H NMR Spectrum of methyl (2-amino-3-cyano-7-methylquinolin-4-yl)-*L*-phenylalaninate (4b)

**Figure S28.** ^13^C NMR Spectrum of methyl (2-amino-3-cyano-7-methylquinolin-4-yl)-L-phenylalaninate (4b)

**Figure S29.** *^1^H NMR Spectrum of methyl (2-amino-3-cyano-7-floroquinolin-4-yl)-L-phenylalaninate (4c)*

**Figure S30.** *^13^C NMR Spectrum of Methyl (2-amino-3-cyano-7-floroquinolin-4-yl)-L-phenylalaninate (4c)*

**Figure S31.** *^1^H NMR Spectrum of methyl (2-amino-7-chloro-3-cyanoquinolin-4-yl)-L-phenylalaninate (4d)*

**Figure S32**. *^13^C NMR Spectrum of methyl (2-amino-7-chloro-3-cyanoquinolin-4-yl)-L-phenylalaninate (4d)*

**Figure S33.** *^1^H NMR Spectrum of methyl (2-amino-6-chloro-3-cyanoquinolin-4-yl)-L-phenylalaninate (4e)*

**Figure S34.** ^13^C NMR Spectrum of *methyl (2-amino-6-chloro-3-cyanoquinolin-4-yl)-L-phenylalaninate (4e)*

**Figure S35.** *^1^H NMR Spectrum of methyl (2-amino-6-bromo-3-cyanoquinolin-4-yl)-L-phenylalaninate (4f)*

**Figure S36.** *^13^C NMR Spectrum of methyl (2-amino-6-bromo-3-cyanoquinolin-4-yl)-L-phenylalaninate (4f)*

**
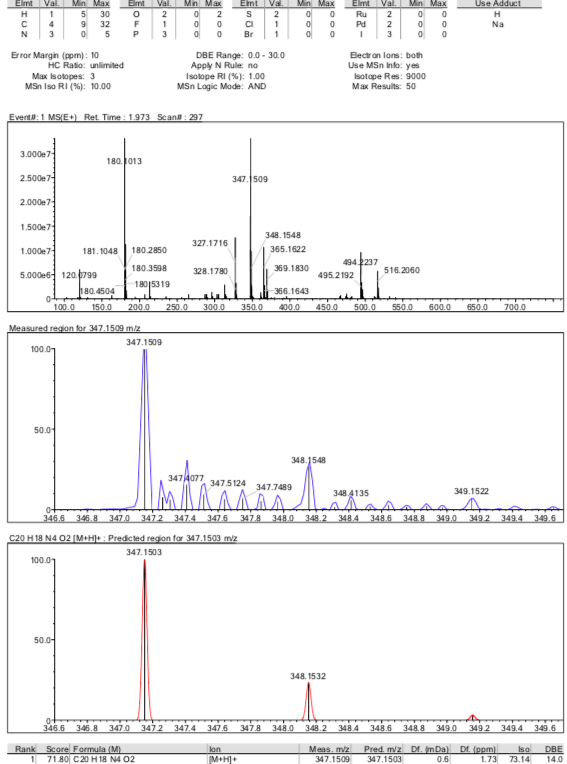
**

**Figure S37.** *HRMS spectrum of* *methyl (2-amino-3-cyanoquinolin-4-yl)-L-phenylalaninate (4a)*

**
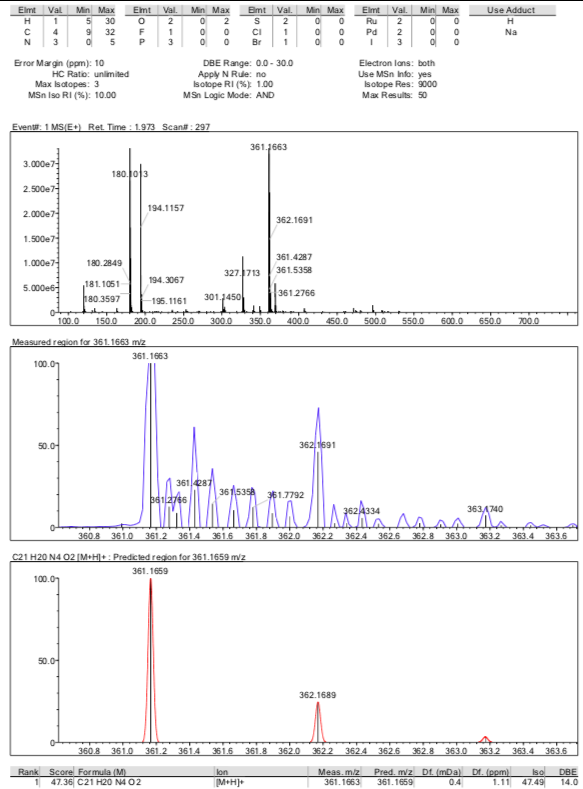
**

**Figure S38.** *HRMS spectrum of methyl (2-amino-3-cyano-7-methylquinolin-4-yl)-L-phenylalaninate (4b)*

**
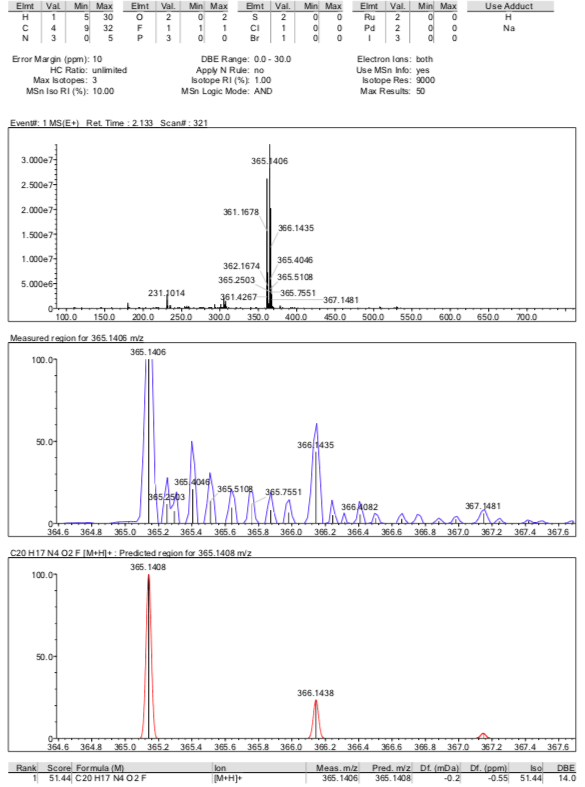
**

**Figure S39.** *HRMS Spectrum* *methyl (2-amino-3-cyano-7-floroquinolin-4-yl)-L-phenylalaninate (4c)*

**
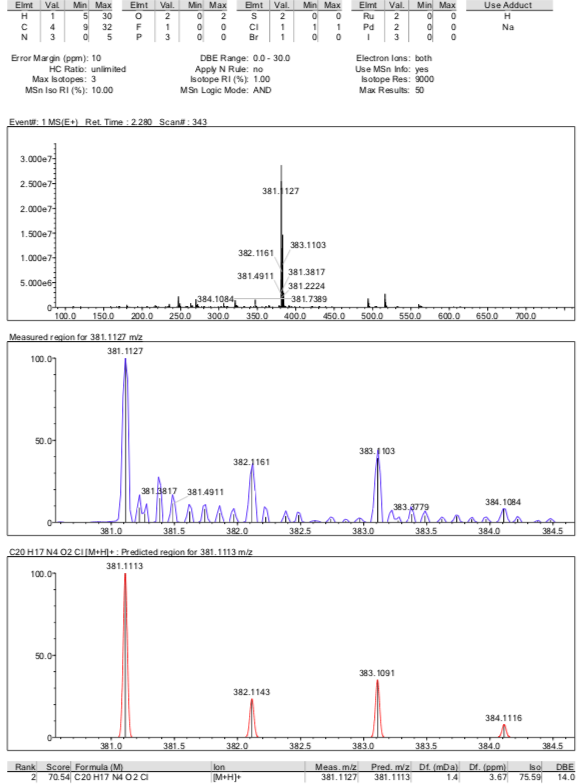
**

**Figure S40.** *HRMS Spectrum of* *Methyl (2-amino-7-chloro-3-cyanoquinolin-4-yl)-L-phenylalaninate (4d)*

**
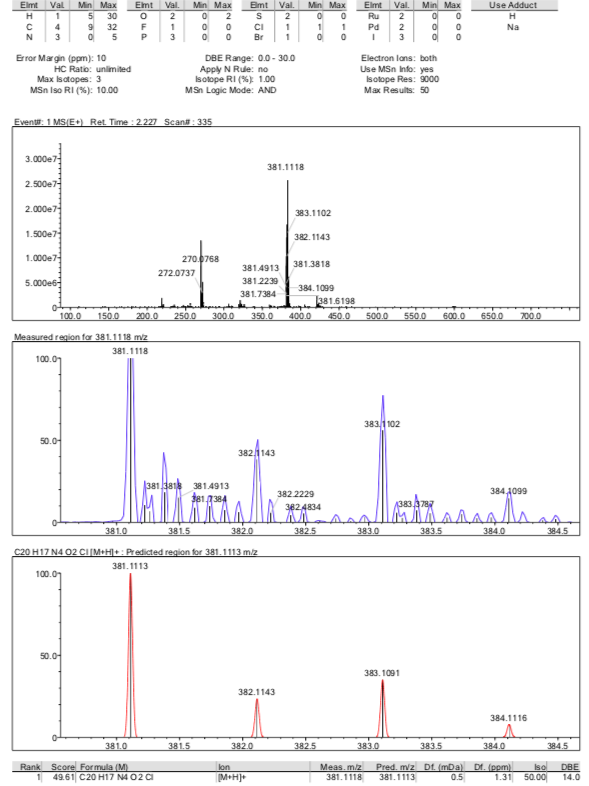
**

**Figure S41.** *HRMS Spectrum of* *Methyl (2-amino-6-chloro-3-cyanoquinolin-4-yl)-L-phenylalaninate (4e)*

**
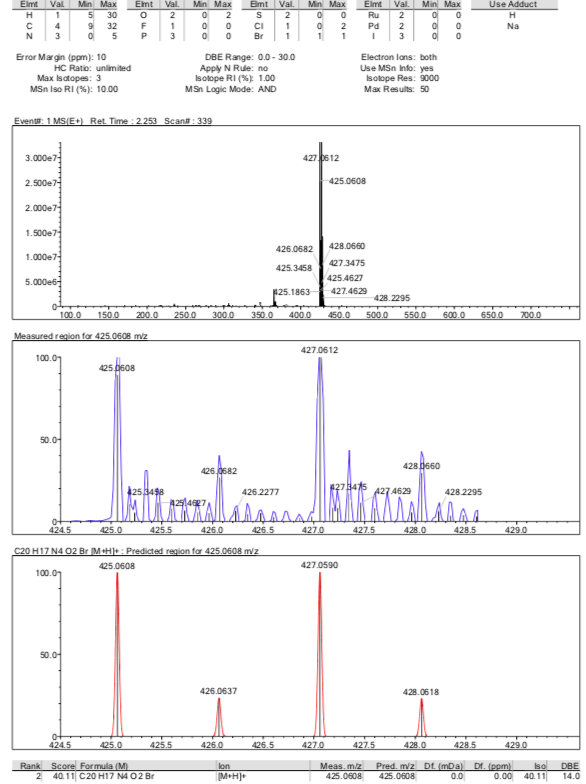
**

**Figure S42.** *HRMS Spectrum of Methyl (2-amino-6-bromo-3-cyanoquinolin-4-yl)-L-phenylalaninate (4f)*


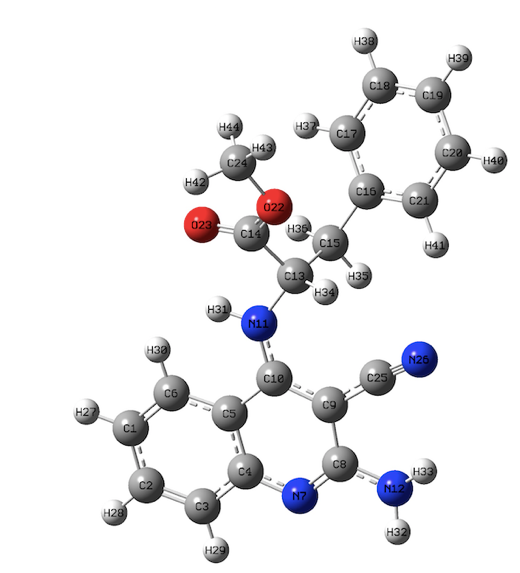


**Figure S43.** *3D view of Compound 4a obtained from GaussView 5.0.8*

**Table S1.** The calculated ^13^C NMR ve ^1^H NMR data for compound 4a in DMSO

| **Atom number** | **Absolute Shielding** | **Chemical Shift** | **Atom number** | **Absolute Shielding** | **Chemical Shift** |
| --- | --- | --- | --- | --- | --- |
| **1** | 74.1335 | 127.2163 | **25** | 74.3666 | 126.9832 |
| **2** | 63.5655 | 137.7843 | **27** | 24.4542 | 7.5623 |
| **3** | 67.6623 | 133.6875 | **28** | 24.0385 | 7.9780 |
| **4** | 47.1767 | 154.1731 | **29** | 24.2114 | 7.8051 |
| **5** | 80.4072 | 120.9426 | **30** | 23.8167 | 8.1998 |
| **6** | 74.6373 | 126.7125 | **31** | 25.2427 | 6.7738 |
| **8** | 38.1383 | 163.2115 | **32** | 27.1681 | 4.8484 |
| **9** | 121.8598 | 79.4900 | **33** | 26.8082 | 5.2083 |
| **10** | 45.1548 | 156.1950 | **34** | 26.3236 | 5.6929 |
| **13** | 131.2884 | 70.0614 | **35** | 27.6444 | 4.3721 |
| **14** | 23.6661 | 177.6837 | **36** | 29.1106 | 2.9059 |
| **15** | 147.4080 | 53.9418 | **37** | 24.4695 | 7.5470 |
| **16** | 59.5720 | 141.7778 | **38** | 24.2035 | 7.8130 |
| **17** | 67.1831 | 134.1667 | **39** | 24.1687 | 7.8478 |
| **18** | 68.4774 | 132.8724 | **40** | 24.1709 | 7.8456 |
| **19** | 69.2727 | 132.0771 | **41** | 23.6660 | 8.3505 |
| **20** | 67.8351 | 133.5147 | **42** | 28.0298 | 3.9867 |
| **21** | 67.5817 | 133.7681 | **43** | 28.4706 | 3.5459 |
| **24** | 140.5603 | 60.7895 | **44** | 28.2222 | 3.7943 |


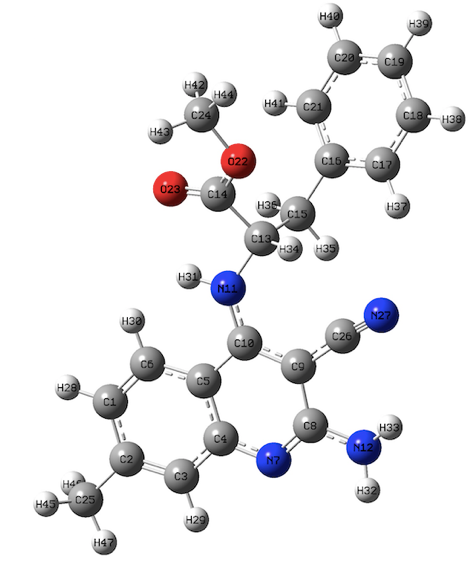


**Figure S44.** *3D view of Compound 4b obtained from GaussView 5.0.8*

**Table S2.** The calculated ^13^C NMR ve ^1^H NMR data for compound 4b in DMSO

| **Atom number** | **Absolute Shielding** | **Chemical Shift** | **Atom number** | **Absolute Shielding** | **Chemical Shift** |
| --- | --- | --- | --- | --- | --- |
| **1** | 73.4491 | 127.9007 | **28** | 24.4826 | 7.5339 |
| **2** | 52.1407 | 149.2091 | **29** | 24.5899 | 7.4266 |
| **3** | 69.6557 | 131.6941 | **30** | 23.9976 | 8.0189 |
| **4** | 47.0220 | 154.3278 | **31** | 25.4595 | 6.5570 |
| **5** | 82.9312 | 118.4186 | **32** | 27.3597 | 4.6568 |
| **6** | 75.6314 | 125.7184 | **33** | 26.7384 | 5.2781 |
| **8** | 40.2070 | 161.1428 | **34** | 26.1639 | 5.8526 |
| **9** | 123.0888 | 78.2610 | **35** | 27.7917 | 4.2248 |
| **10** | 45.5223 | 155.8275 | **36** | 29.2415 | 2.7750 |
| **13** | 132.3625 | 68.9873 | **37** | 23.5547 | 8.4618 |
| **14** | 23.3284 | 178.0214 | **38** | 24.1904 | 7.8261 |
| **15** | 149.5016 | 51.8482 | **39** | 24.2708 | 7.7457 |
| **16** | 59.3036 | 142.0462 | **40** | 24.2686 | 7.7479 |
| **17** | 67.5652 | 133.7846 | **41** | 24.5403 | 7.4762 |
| **18** | 68.2121 | 133.1377 | **42** | 28.2351 | 3.7814 |
| **19** | 69.8027 | 131.5471 | **43** | 27.8665 | 4.1500 |
| **20** | 68.4500 | 132.8998 | **44** | 28.4508 | 3.5657 |
| **21** | 67.4364 | 133.9134 | **45** | 29.2167 | 2.7998 |
| **24** | 140.1715 | 61.1783 | **46** | 29.2003 | 2.8162 |
| **25** | 169.5017 | 31.8481 | **47** | 29.7760 | 2.2405 |
| **26** | 76.6379 | 124.7119 |  |  |  |


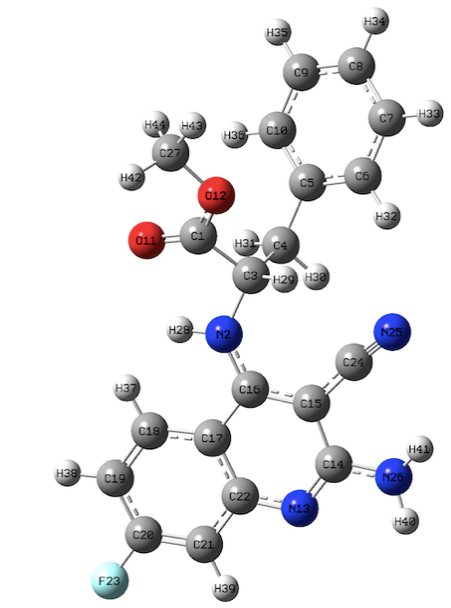


**Figure S45.** *3D view of Compound 4c obtained from GaussView 5.0.8*

**Table S3.** The calculated ^13^C NMR ve ^1^H NMR data for compound 4c in DMSO

| **Atom number** | **Absolute Shielding** | **Chemical Shift** | **Atom number** | **Absolute Shielding** | **Chemical Shift** |
| --- | --- | --- | --- | --- | --- |
| **1** | 23.4158 | 177.9340 | **27** | 140.0741 | 61.2757 |
| **3** | 132.3119 | 69.0379 | **28** | 25.4157 | 6.6008 |
| **4** | 149.4283 | 51.9215 | **29** | 26.1662 | 5.8503 |
| **5** | 59.4199 | 141.9299 | **30** | 27.7782 | 4.2383 |
| **6** | 67.5859 | 133.7639 | **31** | 29.2233 | 2.7932 |
| **7** | 68.1972 | 133.1526 | **32** | 23.5603 | 8.4562 |
| **8** | 69.7515 | 131.5983 | **33** | 24.1883 | 7.8282 |
| **9** | 68.4266 | 132.9232 | **34** | 24.2668 | 7.7497 |
| **10** | 67.4083 | 133.9415 | **35** | 24.2648 | 7.7517 |
| **14** | 39.7694 | 161.5804 | **36** | 24.5364 | 7.4801 |
| **15** | 123.2199 | 78.1299 | **37** | 23.8749 | 8.1416 |
| **16** | 45.5506 | 155.7992 | **38** | 24.7356 | 7.2809 |
| **17** | 83.5391 | 117.8107 | **39** | 24.8469 | 7.1696 |
| **18** | 72.6667 | 128.6831 | **40** | 27.2330 | 4.7835 |
| **19** | 84.9173 | 116.4325 | **41** | 26.6047 | 5.4118 |
| **20** | 30.6867 | 170.6631 | **42** | 27.8589 | 4.1576 |
| **21** | 84.6405 | 116.7093 | **43** | 28.4445 | 3.5720 |
| **22** | 44.9910 | 156.3588 | **44** | 28.2289 | 3.7876 |
| **24** | 76.8786 | 124.4712 |  |  |  |


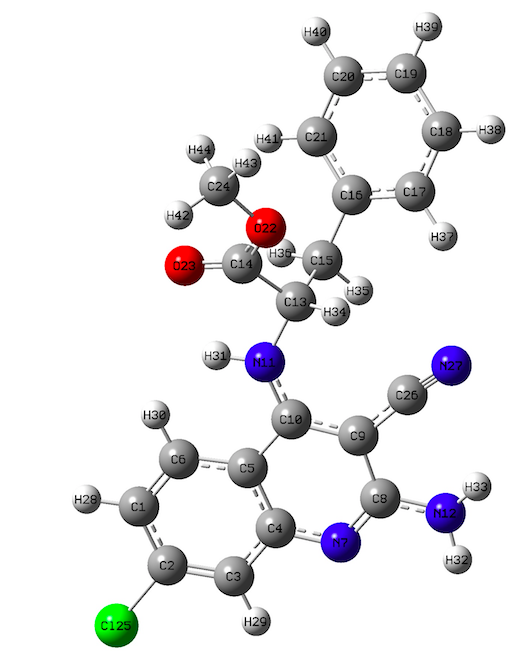


**Figure S46.** *3D view of Compound 4d obtained from GaussView 5.0.8*

**Table S4.** The calculated ^13^C NMR ve ^1^H NMR data for compound 4d in DMSO

| **Atom number** | **Absolute Shielding** | **Chemical Shift** | **Atom number** | **Absolute Shielding** | **Chemical Shift** |
| --- | --- | --- | --- | --- | --- |
| **1** | 74.6179 | 126.7319 | **26** | 77.1262 | 124.2236 |
| **2** | 50.2374 | 151.1124 | **28** | 24.6253 | 7.3912 |
| **3** | 70.3373 | 131.0125 | **29** | 24.5136 | 7.5029 |
| **4** | 46.1666 | 155.1832 | **30** | 24.0056 | 8.0109 |
| **5** | 81.6955 | 119.6543 | **31** | 25.4259 | 6.5906 |
| **6** | 73.8746 | 127.4752 | **32** | 27.2090 | 4.8075 |
| **8** | 39.7883 | 161.5615 | **33** | 26.5817 | 5.4348 |
| **9** | 122.6479 | 78.7019 | **34** | 26.2147 | 5.8018 |
| **10** | 45.6176 | 155.7322 | **35** | 27.7810 | 4.2355 |
| **13** | 132.2822 | 69.0676 | **36** | 29.2127 | 2.8038 |
| **14** | 23.4814 | 177.8684 | **37** | 23.6008 | 8.4157 |
| **15** | 149.2533 | 52.0965 | **38** | 24.1959 | 7.8206 |
| **16** | 59.5327 | 141.8171 | **39** | 24.2698 | 7.7467 |
| **17** | 67.5776 | 133.7722 | **40** | 24.2669 | 7.7496 |
| **18** | 68.1816 | 133.1682 | **41** | 24.5432 | 7.4733 |
| **19** | 69.7131 | 131.6367 | **42** | 27.8592 | 4.1573 |
| **20** | 68.3973 | 132.9525 | **43** | 28.4530 | 3.5635 |
| **21** | 67.4619 | 133.8879 | **44** | 28.2484 | 3.7681 |
| **24** | 140.0759 | 61.2739 |  |  |  |


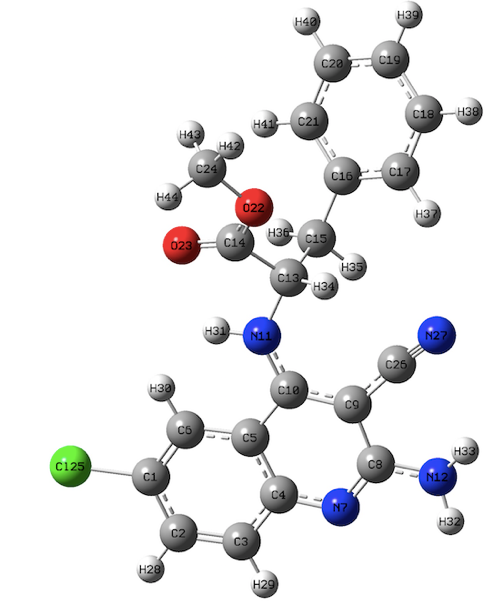


**Figure S47.** *3D view of Compound 4e obtained from GaussView 5.0.8*

**Table S5.** The calculated ^13^C NMR ve ^1^H NMR data for compound 4e in DMSO

| **Atom number** | **Absolute Shielding** | **Chemical Shift** | **Atom number** | **Absolute Shielding** | **Chemical Shift** |
| --- | --- | --- | --- | --- | --- |
| **1** | 61.1517 | 140.1981 | **26** | 77.1672 | 124.1826 |
| **2** | 64.3049 | 137.0449 | **28** | 24.2656 | 7.7509 |
| **3** | 67.9965 | 133.3533 | **29** | 24.3895 | 7.6270 |
| **4** | 48.1760 | 153.1738 | **30** | 24.1399 | 7.8766 |
| **5** | 80.0131 | 121.3367 | **31** | 25.5042 | 6.5123 |
| **6** | 75.3942 | 125.9556 | **32** | 27.2397 | 4.7768 |
| **8** | 40.1845 | 161.1653 | **33** | 26.6133 | 5.4032 |
| **9** | 122.3276 | 79.0222 | **34** | 26.1969 | 5.8196 |
| **10** | 46.1929 | 155.1569 | **35** | 27.7759 | 4.2406 |
| **13** | 132.2648 | 69.0850 | **36** | 29.1962 | 2.8203 |
| **14** | 23.5038 | 177.8460 | **37** | 23.5856 | 8.4309 |
| **15** | 149.1816 | 52.1682 | **38** | 24.1928 | 7.8237 |
| **16** | 59.5249 | 141.8249 | **39** | 24.2665 | 7.7500 |
| **17** | 67.5874 | 133.7624 | **40** | 24.2625 | 7.7540 |
| **18** | 68.1945 | 133.1553 | **41** | 24.5320 | 7.4845 |
| **19** | 69.7089 | 131.6409 | **42** | 28.4425 | 3.5740 |
| **20** | 68.3971 | 132.9527 | **43** | 28.2356 | 3.7809 |
| **21** | 67.4371 | 133.9127 | **44** | 27.8493 | 4.1672 |
| **24** | 140.0380 | 61.3118 |  |  |  |

| 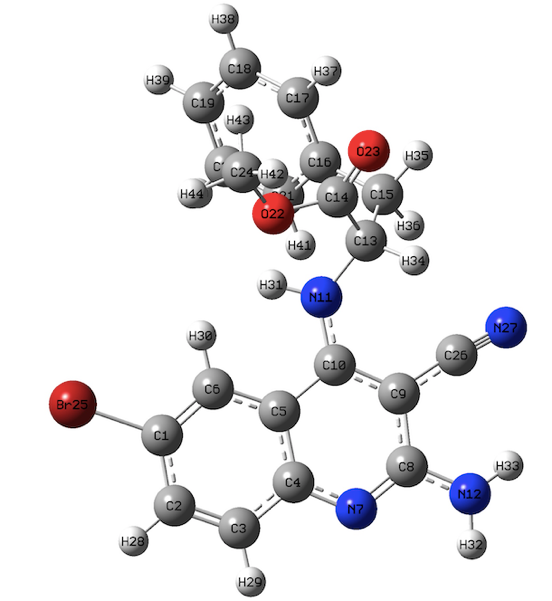 | 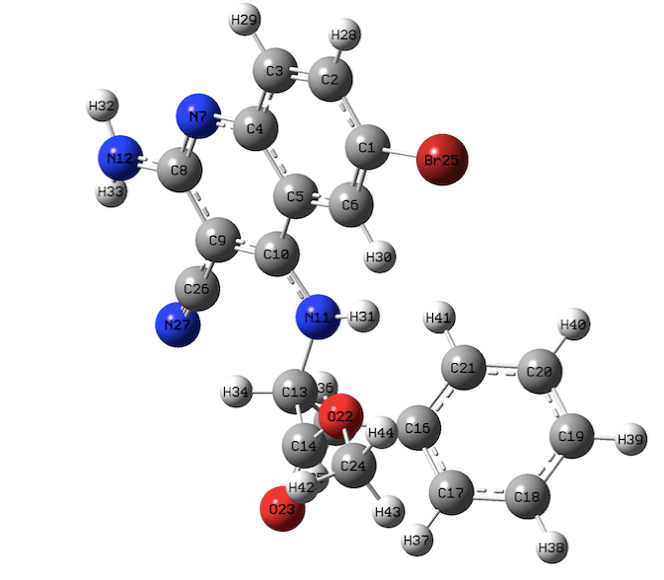 |
| --- | --- |

**Figure S48.** *3D view of Compound 4f obtained from GaussView 5.0.8*

**Table S6.** The calculated ^13^C NMR ve ^1^H NMR data for compound 4f in DMSO

| **Atom number** | **Absolute Shielding** | **Chemical Shift** | **Atom number** | **Absolute Shielding** | **Chemical Shift** |
| --- | --- | --- | --- | --- | --- |
| **1** | 62.8960 | 138.4538 | **26** | 78.2990 | 123.0508 |
| **2** | 61.8129 | 139.5369 | **28** | 24.2079 | 7.8086 |
| **3** | 67.6585 | 133.6913 | **29** | 24.3737 | 7.6428 |
| **4** | 47.9379 | 153.4119 | **30** | 24.3493 | 7.6672 |
| **5** | 79.2356 | 122.1142 | **31** | 26.0726 | 5.9439 |
| **6** | 72.6164 | 128.7334 | **32** | 27.2944 | 4.7221 |
| **8** | 39.2987 | 162.0511 | **33** | 26.7033 | 5.3132 |
| **9** | 121.8210 | 79.5288 | **34** | 26.1966 | 5.8199 |
| **10** | 45.7618 | 155.5880 | **35** | 28.3592 | 3.6573 |
| **13** | 131.5911 | 69.7587 | **36** | 28.3225 | 3.6940 |
| **14** | 27.5712 | 173.7786 | **37** | 24.4033 | 7.6132 |
| **15** | 147.8158 | 53.5340 | **38** | 24.1951 | 7.8214 |
| **16** | 60.8878 | 140.4620 | **39** | 24.2383 | 7.7782 |
| **17** | 67.6839 | 133.6659 | **40** | 24.2908 | 7.7257 |
| **18** | 68.0545 | 133.2953 | **41** | 24.4139 | 7.6026 |
| **19** | 69.3180 | 132.0318 | **42** | 27.8571 | 4.1594 |
| **20** | 68.4693 | 132.8805 | **43** | 27.9474 | 4.0691 |
| **21** | 66.3799 | 134.9699 | **44** | 28.0748 | 3.9417 |
| **24** | 139.0130 | 62.3368 |  |  |  |
